# Sex and racial/ethnic disparities in motor vehicle crash fatalities among U.S. Teens aged 15–19 years, 2015–2023

**DOI:** 10.3389/fpubh.2026.1760179

**Published:** 2026-04-01

**Authors:** Dominique M. Rose, Enas Alshaikh, Saroj Bista, Motao Zhu, Cynthia J. Sieck, Jingzhen Yang

**Affiliations:** 1 Center for Injury Research and Policy, Abigail Wexner Research Institute at Nationwide Children’s Hospital, Columbus, OH, United States; 2 Department of Pediatrics, College of Medicine, The Ohio State University, Columbus, OH, United States; 3 Center for Health Equity, Dayton Children’s Hospital, Dayton, OH, United States

**Keywords:** fatal crash involvement, fatality analysis reporting system, injury prevention, race, ethnicity, disparity, teen driver

## Abstract

**Introduction:**

Motor vehicle crashes (MVCs) remain the leading cause of injury and death among U.S. teens. Although disparities in crash fatalities by sex and race/ethnicity are well documented, few studies have focused specifically on teens and teen drivers. This study examined national trends in MVC fatality rates among teens aged 15–19 years in the United States by sex and race/ethnicity.

**Methods:**

We conducted a retrospective analysis using 2015–2023 data from the Fatality Analysis Reporting System (FARS). Fatalities included all teens aged 15–19 who died in MVCs, regardless of role (driver, passenger, or non-driver). Negative binomial regression models estimated rate ratios (RRs) and 95% confidence intervals (CIs) for annual fatality rates per 100,000 population, adjusted for sex, race/ethnicity, and COVID-19 period (pre-, during-, and post-pandemic).

**Results:**

Among 22,997 teen fatalities, 67% were male, 52% White, 15% Black, and 22% Hispanic; 55% were drivers. Male teens (RR = 1.8, 95% CI: 1.4–2.4) and male teen drivers (RR = 2.6, 95% CI: 1.9–3.4) had significantly higher fatality rates than females. American Indian/Alaska Native teens had the highest fatality rates across all racial groups. Black male teens had higher rates than White male teens, although Black teen drivers in both sexes had significantly lower MVC fatality rates than White teen drivers. No significant differences were observed between Hispanic and non-Hispanic groups. Fatality rates increased during the COVID-19 pandemic, peaking in 2021, though changes across periods were not statistically significant.

**Discussion:**

Male and American Indian/Alaska Native teens remain disproportionately affected by MVC-related deaths in the United States, underscoring persistent disparities by sex and race/ethnicity. In contrast, lower fatality rates observed among Black teen drivers relative to White teen drivers may reflect differences in licensure rates, vehicle access, and driving exposure. Targeted interventions that promote equitable access to driver education, such as driving school vouchers, and providing financial support for vehicle maintenance and safety checks are essential to reducing these disparities and improving safety among high-risk teen populations.

## 1 Introduction

Despite significant improvements in traffic safety in recent years, motor vehicle crashes (MVCs) remain the leading cause of injury and death among teens in the United States (U.S.) (1, 2). Statistics showed that teens aged 16 to 19 are nearly three times more likely to be involved in a fatal crash per mile driven compared to drivers of other age groups, although they only drive half the annual miles of adult drivers (1–3). The elevated injuries and fatalities related to MVCs among teen drivers stem from a complex interplay of individual maturity, vehicle dynamics, and environmental factors (4). Developmental factors, such as incomplete brain maturation, also affect teen drivers’ judgment, reaction times, and hazard perception, making them more vulnerable on the road (4, 5). Significantly, crash risks and severities are not uniform across all populations of young drivers (6). Teens who are male or from lower socioeconomic backgrounds and minority groups are disproportionately affected by MVCs (7).

Numerous studies have documented that racial and ethnic minority groups and economically disadvantaged populations are disproportionately represented in traffic crashes, deaths, and injuries (7–9). These disparities persist among vulnerable teen populations, potentially due to inequities in access to driver education, safer vehicles, and driving opportunities, which contribute to differences in driving exposure and elevated risks of crash-related injury and death (10, 11). Analyses using the Fatality Analysis Reporting System (FARS) have shown that Black men were at an increased risk of dying when traveling in motor vehicles while Hispanic men had an increased risk of dying as motor vehicle occupants (8). Similarly, a recent report from the Governors Highway Safety Association using 2015–2019 FARS data identified substantial racial and ethnic disparities in traffic fatalities (12), with highest rates among American Indian/Alaskan Native and Black Americans and lowest rates among Asian Americans. Despite this evidence, important gaps remain. Most national studies have examined racial and ethnic disparities in fatalities among drivers across all age groups (13–15), rather than focusing specifically on teens, a population with uniquely high crash risk. Additionally, while numerous studies have investigated racial disparities in risky driving behaviors among teen drivers (16–18), few national analyses have specifically explored how sex, race/ethnicity jointly related to MVC fatality rates among U.S. teens and teen drivers (19, 20). Furthermore, although travel volume declined during COVID-19 lockdowns, traffic fatalities increased during this period (21–24), likely due to higher average vehicle speeds, yet national evaluations of teen fatality patterns during COVID-19 pandemic remain limited.

To address these gaps, this study aimed to (1) examine the MVC fatality rates among teens and teen drivers aged 15 to 19 years by sex and race/ethnicity using data from the 2015–2023 FARS; and (2) investigate the associations of sex, and race/ethnicity with MVC fatality rates among teens and teen drivers aged 15–19 years. We hypothesized that male teens and teen drivers would have higher fatality rates per 100,000 population than female teens and teen drivers, and that the fatality rates would differ across race/ethnicity groups; American Indian/Alaskan Native teens involved in fatal MVCs would have the highest fatality rates while Asian teens involved in fatal MVCs would have the lowest annual fatality rate. Compared to White teen drivers, Black teen drivers would have lower fatality rates.

## 2 Materials and methods

### 2.1 Data and study population

Data used for this current study were sourced from the FARS for the years 2015 through 2023. FARS data, managed by National Highway Traffic Safety Administration (NHTSA), includes all fatal crashes within the United States and compiles crashes if at least one person (either a motorist or a non-motorist) involved in the crash dies within 30 consecutive days from the time of the crash. For the purpose of this study, we limited our analysis to the crashes involving teens aged 15–19 years (“all teens”) who died in a crash, regardless of vehicle occupancy status (driver, passenger, or non-driver) or pedestrian status. In addition, we conducted a sub-group analysis among teen drivers aged 15–19 years (“teen drivers”) who died in crashes as drivers regardless of vehicle type or crash complexity. This study was deemed exempt from Institutional Review Board approval at our institution (STUDY00004504).

### 2.2 Explanatory factors

Main explanatory factors were sex and race/ethnicity of teens. Biological sex (categorized as male or female) was available in the FARS data for all teens involved in a fatal crash. Data on race and ethnicity were only available in the FARS for teens who died in a crash based on the information from death certificates. In this study, we categorized teens’ race/ethnicity into 7 groups using classification guidelines provided by Governors Highway Safety Association (12) (1) non-Hispanic/Unknown White (“White”), (2) non-Hispanic/Unknown Black (“Black”), (3) non-Hispanic/Unknown American Indian or Alaska Native (“American Indian or Alaska Native”), (4) non-Hispanic/Unknown Asian (“Asian”), (5) non-Hispanic/Unknown Native Hawaiian and other Pacific Islander (“‘Native Hawaiian and other Pacific Islander”); (6) non-Hispanic/Unknown Two or More Races (“Two or More Races”), and (7) Hispanic (any race). For simplicity, we omit the prefix “non-Hispanic/Unknown” and refer to these categories by race/ethnicity names in the text.

### 2.3 Outcomes

We included two outcomes in our analysis: (1) annual MVC fatalities rate per 100,000 population of teens aged 15–19 years (all teen fatalities), and (2) the annual MVC fatalities rate per 100,000 population of teen drivers aged 15–19 years who died in a crash, from 2015 through 2023. For each outcome, we calculated yearly rates from 2015 through 2023 by dividing the number of relevant fatalities (all teens or teen drivers) in that year by the estimated population of 15–19 years olds in the U.S. during that year, then multiplying by 100,000. We then computed the average of these annual rates across the full study period, as well as within the three distinct intervals: pre-COVID-19 (2015–2019), during COVID-19 (2020–2021), and post-COVID-19 (2022–2023). Population denominators were estimated using data from the American Community Survey (ACS, 2019, 2022–2024), stratified by year, sex, race/ethnicity, and their cross-classifications to obtain subgroup populations. Race/ethnicity specific fatality rates were derived similarly using subgroup-level numerators and corresponding subgroup population denominators.

### 2.4 Statistical analysis

We used negative binomial regressions (NB) to examine overall annual MVC fatality rates among teens aged 15–19 years, as well as rates stratified by sex, race/ethnicity, and COVID-19 period, accounting for overdispersion. The log population denominator was used as an offset variable. The study period was categorized as pre-COVID-19 (2015–2019), during COVID-19 (2020–2021), and post-COVID-19 (2022–2023), and this variable was included in adjusted regression models to account for broad pandemic-related influences. We estimated unadjusted rate ratios (RRs) and 95% confidence intervals (CIs) for MVC fatalities by sex, race/ethnicity, and COVID-19 period, with a RR greater than 1.0 indicating increased risk and a RR less than 1.0 indicating reduced risk relative to the reference group. To compare MVC fatality rates across race/ethnicity groups within each sex, we conducted additional analyses by including COVID-19 period, sex, race/ethnicity, and their interaction terms in the regression models. Tukey–Kramer adjustment was used for multiple comparisons. We applied the same analytic approaches to the subgroup of teens who died as drivers. Overdispersion was assessed using the Pearson *χ*^2^/df statistic, and model fit was evaluated with standardized residual plots and plots comparing observed versus predicted values (25, 26). All analyses were conducted in SAS, version 9.4., with a 2-sided *α* level of 0.05 for statistical significance.

## 3 Results

### 3.1 Number, annual rates, and unadjusted rate ratios of motor vehicle crash fatalities

A total of 22,997 teens aged 15 to 19 died in fatal MVCs between 2015 and 2023. Of these, 67.4% (*n* = 15,510) were males, 52.1% (*n* = 11,978) were White, 15.3% (*n* = 3,512) were Black, and 54.8% (*n* = 12,597) died as drivers (Table 1). The overall annual MVC fatalities per 100,000 population was 11.9 (95% CI: 11.4–12.4) among teens and 6.5 (95% CI: 6.2–6.9) among teen drivers, respectively. The annual fatality rate per 100,000 population was 15.7 (95% CI: 14.9–16.6) for male teens and 7.9 (95% CI: 7.5–8.3) for female teens. This indicates that male teens were 1.8 times more likely to die in fatal MVCs compared to female teens (RR = 1.8, 95% CI: 1.4–2.4) (Table 1). Among teen drivers, the sex disparity was even greater: male drivers had 2.6 times the fatality rate of female drivers (RR = 2.6, 95% CI: 1.9–3.4). The annual rates were 9.5 (95% CI: 8.8–10.1) for male drivers and 3.5 (95% CI: 3.2–3.7) for female drivers per 100,000 population.

**Table 1:** Fatality rate among teens and teen drivers aged 15–19 by sex and race/ethnicity due to motor vehicle crashes in the United States, 2015–2023.

Variable	All teens			Teen drivers		
	Deaths(%)^a^	Annual rate/100,000 population	Unadjusted rate ratio (RR)	Deaths (%)^a^	Annual rate/100,000 population	Unadjusted rate ratio (RR)
		(95% CI)	(95% CI)		(95% CI)	(95% CI)
Total	22,997 (100.0)	11.9 (11.4–12.4)		12,597 (100.0)	6.5 (6.2–6.9)	
Sex^a^						
Female	7,463 (32.5)	7.9 (7.5–8.3)	Ref	3,260 (25.9)	3.5 (3.2–3.7)	Ref
Male	15,510 (67.4)	15.7 (14.9–16.6)	1.8 (1.4–2.4)	9,328 (74.0)	9.5 (8.8–10.1)	2.6 (1.9–3.4)
Unknown/missing	24 (0.1)			9 (0.1)		
Race/ethnicity^b^						
Non-Hispanic White	11,978 (52.1)	11.9 (11.3–12.5)	Ref	7,294 (57.9)	7.2 (6.9–7.6)	Ref
Non-Hispanic Black	3,512 (15.3)	13.2 (12.0–14.4)	1.1 (0.9–1.4)	1,542 (12.2)	5.8 (5.0–6.6)	0.8 (0.6–1.1)
Non-Hispanic American Indian and Alaska native	415 (1.8)	25.7 (22.7–28.8)	2.2 (1.7–2.8)	199 (1.6)	12.4 (10.7–13.9)	1.7 (1.2–2.5)
Non-Hispanic Asian	376 (1.6)	3.8 (3.2–4.3)	0.3 (0.2–0.4)	171 (1.4)	1.7 (1.4–2.1)	0.2 (0.2–0.3)
Non-Hispanic native Hawaiian and other Pacific Islander	32 (0.1)	8.6 (4.3–12.9)	0.7 (0.5–1.1)	11 (0.1)	2.9 (1.6–4.3)	0.4 (0.2–0.8)
Two or more races	155 (0.7)	2.2 (1.6–2.7)	0.2 (0.1–0.2)	71 (0.6)	1.0 (0.8–1.2)	0.1 (0.1–0.2)
Hispanic^c^	5,044 (21.9)	10.8 (10.2–11.5)	1.0 (0.7–1.5)	2,551 (20.3)	5.5 (5.0–5.9)	1.0 (0.7–1.6)
Unknown/missing	1,485 (6.5)			758 (6.0)		
COVID-19						
Pre-COVID-19 (2015–2019)	12,250 (53.3)	11.6 (10.8–12.5)	Ref	6,600 (52.4)	6.3 (5.9–6.7)	Ref
During COVID-19 (2020–2021)	5,312 (23.1)	12.3 (11.1–13.6)	1.0 (0.7–1.4)	2,927 (23.2)	6.8 (5.8–7.8)	1.1 (0.7–1.6)
Post-COVID-19 (2022–2023)	5,435 (23.6)	12.3 (11.7–13.0)	1.0 (0.7–1.5)	3,070 (24.4)	7.0 (6.6–7.3)	1.2 (0.8–1.7)

CI, Confidence Interval; ref, reference.

^a^Column percentages may not sum exactly to 100% due to rounding error.

^b^Tukey–Kramer adjustment was used for multiple comparisons.

^c^Hispanic teens may be of any race; Reference group non-Hispanic/Unknown.

American Indian or Alaska Native teens had the highest annual MVC fatality rate (25.7 per 100,000; 95% CI: 22.7–28.8), followed by Black teens (13.2; 95% CI: 12.0–14.4) and White teens (11.9; 95% CI: 11.3–12.5) (Table 1). Among teen drivers, American Indian or Alaska Native teen drivers remained the highest rate (12.4; 95% CI: 10.7–13.9). In contrast, Black teen drivers had a lower fatality rate than White teen drivers (5.8 vs. 7.2 per 100,000). Compared with White teens, American Indian or Alaska Native teens were more than twice as likely to die in fatal MVCs (RR = 2.2, 95% CI: 1.7–2.8), and their teen drivers showed a similar elevated fatality rate (RR = 1.7, 95% CI: 1.2–2.5). Teens and drivers who were Asian, and of two or more races had significantly lower fatality rates than their White counterparts. Hispanic teens and drivers had fatality rates comparable to non-Hispanic teens and drivers.

### 3.2 Annual MVC fatality rates across pre-, during-, and post-COVID-19 periods

**Figure 1:**
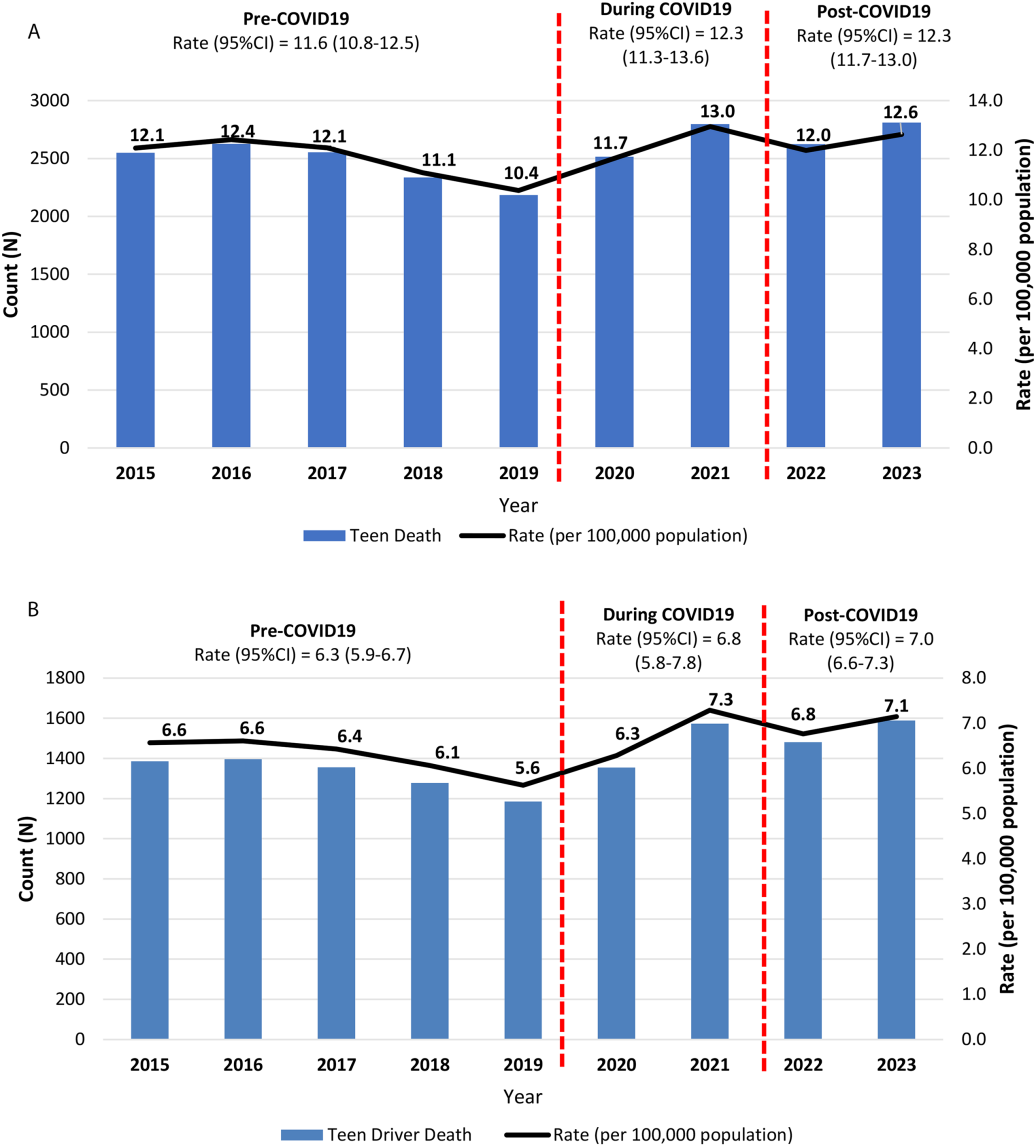
**(A)** Death counts and fatality rates among all teens aged 15–19, due to motor vehicle crashes in the United States, 2015–2023; **(B)** Death counts and fatality rates among teen drivers aged 15–19, due to motor vehicle crashes in the United States, 2015–2023.

The annual fatality rates for teen and teen drivers steadily decreased over the pre-COVID-19 period (2015–2019), reaching the lowest levels in 2019, with 10.4 and 5.6 per 100,000 population for teens and teen drivers, respectively (Figures 1A,B). The annual fatality rates then increased during the COVID-19 period (2020–2021), peaking in 2021 at 13.0 per 100,000 population for teens and 7.3 per 100,000 population for teen drivers. In the post-COVID-19 period (2022–2023), both groups showed gradual declines in fatality rates. However, these differences across the three periods were not statistically significant.

### 3.3 Adjusted fatality rate ratios across sex and race/ethnicity

**Figure 2:**
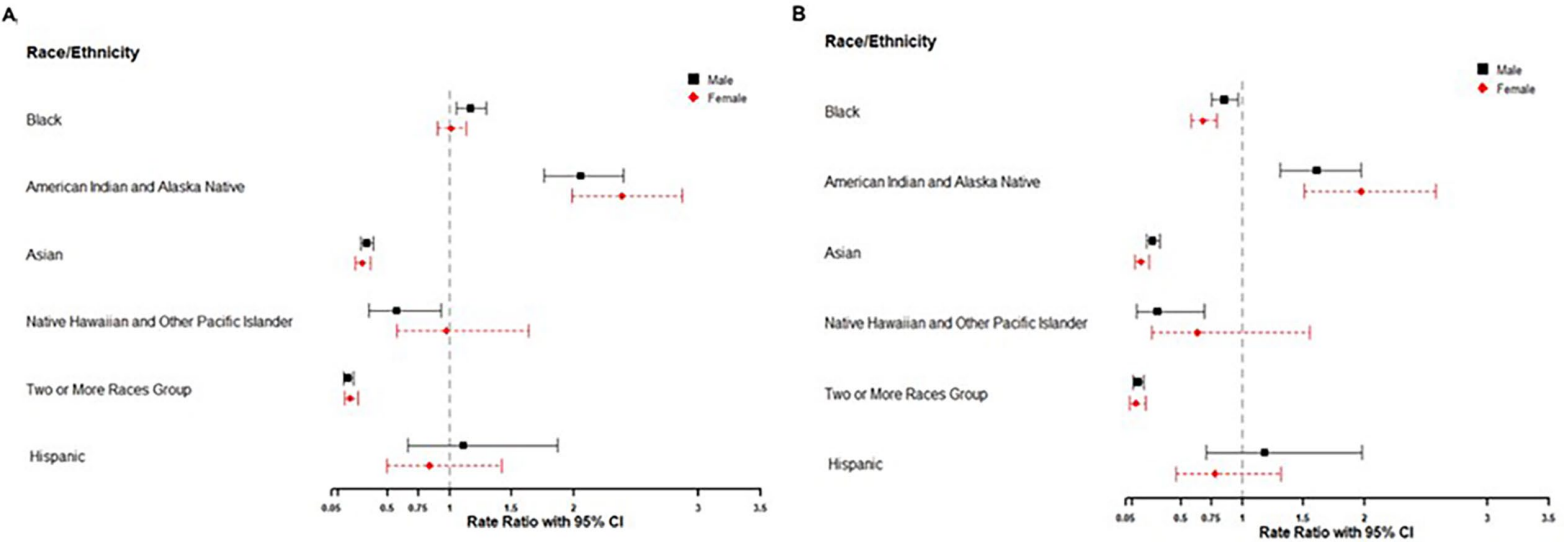
**(A,B)** Motor vehicle fatality rate ratio by race/ethnicity and sex among teens and teen drivers aged 15–19 in the United States, 2015–2023. Cases excluded where sex (all teens = 24; teen drivers = 9) or race/ethnicity (all teens = 1,485; teen drivers = 758) was missing/unknown. All race groups reported were non-Hispanic/unknown ethnicity and Hispanic teens may be of any race. Reference: The reference group for Hispanic was non-Hispanic/unknown. All other race groups used non-Hispanic/unknown White as a reference group. Model adjusted for COVID-19 periods, sex, race/ethnicity, and the interaction term between sex and race/ethnicity.

Black female teens had a motor vehicle fatality rate similar to that of White female teens (RR = 1.0; 95% CI: 0.9–1.1), while Black male teens had a significantly higher fatality rate than White male teens (RR = 1.2; 95% CI: 1.1–1.3) (Figure 2A). In contrast, both Black male (RR = 0.85; 95% CI: 0.75–0.96) and Black female (RR = 0.7; 95% CI: 0.6–0.8) teen drivers had significantly lower fatality rates than their respective White counterparts (Figure 2B). For both sexes, American Indian and Alaska Native teens and teen drivers had fatality rates approximately twice as high as those of White teens. Conversely, Asian and Two or More Races teens and teen drivers (both sexes) experienced significantly lower fatality rates compared with White people. There were no statistically significant differences in fatality rates between Hispanic and non-Hispanic teens or teen drivers for either sex.

## 4 Discussion

This study examined the annual fatality rates per 100,000 population of teen and teen drivers aged 15–19 by sex, and race/ethnicity using data from the 2015–2023 FARS. The main findings indicate that male teens and teen drivers consistently had higher MVC-related fatality rates than females. American Indian or Alaska Native teens and teen drivers experienced the highest rate of MVC-related fatality rates compared with all other racial groups. Fatality rates increased during the COVID-19 pandemic, reaching their highest levels in 2021 for both teens and teen drivers. The observed increase is consistent with prior findings indicating that substantial reductions in traffic volume during COVID-19 lockdowns led to lower roadway congestion, higher travel speeds, and increased risky driving behaviors, particularly speeding, which in turn contributed to greater crash severity and higher fatality despite an overall decline in total crashes (21–24, 27). Our results contribute to the existing literature by demonstrating that MVC-related deaths among teens and teen drivers vary across sex, and race/ethnicity (8, 10, 11, 28, 29). Given that motor vehicle crashes are the leading cause of death for adolescents aged 15–19 in the U.S. (1, 2), these results highlight the need for developing and implementing targeted interventions for different groups of teen drivers, particularly those from minority groups, to minimize MVC-related deaths.

Consistent with previous research findings (11), we found that Black male and female teen drivers had significantly lower MVC-related fatality rates than White teen drivers. However, Black male teens had higher rates than White male teens involved in fatal crashes and no significant difference was observed between Black and White female teens, suggesting that Black teens may be more likely to die as a non-driver or passengers in these incidents. Imai et al. (11) explored fatality rates across racial and ethnic groups of young drivers in North Carolina and found that young Black teen drivers had a lower fatality rate compared to White teen drivers. Tefft et al. (30) found that 37.0% of Black and 29.0% of Hispanic teens attained their driver’s license before the age of 18 compared to 67.0% of White teens, showing a delay in obtaining a driver’s license (31, 32). The lower fatality rate observed among Black teen drivers may reflect a health inequity, such as reduced driving opportunities related to driver’s education and delayed licensure, rather than a true protective effect (31, 33). Even with a driver’s license, Black licensed teen drivers may have less access to a vehicle compared to White teen drivers, potentially contributing to the observed lower fatality rate in this study (34). Given that teen drivers have the highest MVC risk of any age groups (28), and that racial minority teens are disproportionally affected by the risk of crash-related deaths (8), our findings, together with prior research (30, 31), emphasize the critical need for innovative strategies to address racial and socioeconomic disparities in access to driver education and behind-the-wheel training. Limited access to these resources can delay licensure, constrain skill development, and increase crash risk. Community-based and equity-focused approaches, such as subsidized or school-based driver education programs, partnerships with community organizations to provide low-cost behind-the-wheel training, parent engagement and coaching programs for families with limited driving experience, and technology-enabled training and feedback tools, offer promising opportunities to improve access, promote safer driving behaviors, and reduce disparities in licensure outcomes and crash risk among diverse teen populations (35–38).

We observed an increase in MVC-related fatality rates among both teens and teen drivers during the COVID-19 pandemic, with rates peaking in 2021 in the U.S. However, comparison with pre- and post-COVID-19 pandemic years showed no statistically significant differences. This upward trend aligns with previous findings, including reports from the NHTSA (27, 39–41), which documented increases in traffic crashes and crash-related fatalities following the onset of the COVID-19 pandemic. Prior research by Hughes and colleagues (24) also found a higher frequency of severe crashes and crash-related deaths during a 10-week period in 2020, likely driven by reduced traffic congestion and higher highway speeds, particularly among male drivers and those under 25 years of age (24). Despite overall reductions in miles driven and traffic volume, COVID restrictions unintentionally increased the risk of high-speed and severe crashes among young drivers (21, 27, 42, 43). These findings underscore the need for comprehensive strategies to improve teen driver safety. Targeted interventions should promote consistent seat belt use, restrict underage access to alcohol, and strengthen parental involvement and supervision during the first 6 months of independent driving. Addressing structural barriers, such as limited access to driver education, safe vehicles, and training resources among racial and ethnic minority teens, is also critical for reducing disparities in crash risk and improving equity in road safety outcomes.

This study found that male teens and male teen drivers consistently had higher MVC-related fatalities than their female counterparts, with male teen drivers experiencing nearly three times the annual fatality rate of female drivers. These findings align with previous research showing that male teen drivers have the highest rates of crash-related deaths and involvement in fatal crashes across all age groups (10, 28, 44, 45). Prior studies also indicate that crash characteristics differ by sex, with female drivers engaging in fewer risky driving behaviors, and being less frequently exposed to high-risk driving environments compared to males (29, 44, 46). Our study also revealed racial and ethnic disparities in MVC-related fatality rates. American Indian/Alaskan Native teens consistently face higher mortality rates, while Asian teens had lower rates of MVC-related fatalities compared with their White peers, regardless of sex or driver status. Hispanic male and female teen and teen drivers had fatality rates similar to their non-Hispanic counterparts. These patterns, supported by prior research (47, 48), confirm that male and American Indian/Alaskan Native teens and teen drivers are particularly vulnerable to MVC-related fatalities. To reduce crash-related deaths among high-risk teen groups, interventions require coordinated, multi-level strategies that operate at the individual, family, community, and policy levels. At the individual level, skills-based driver education and simulation- or technology-assisted training can improve hazard recognition and decision-making. At the family-level, parent engagement programs can increase supervised practice and reinforce safe driving norms during early licensure. Community-level approaches, including school-based or subsidized driver education, partnerships with community organizations, and access to safe vehicles, can reduce barriers faced by disadvantaged teens. At the policy level, robust Graduated Driver Licensing (GDL) systems, primary enforcement of seat belt and alcohol laws, and restrictions on nighttime driving and teen passengers can further reduce exposure to high-risk conditions (36, 37, 49–51). Collectively, these integrated strategies can reduce crash risk, prompt safe driving behaviors, and address disparities in teen driver safety outcomes (12).

### 4.1 Limitations

Several limitations warrant consideration when interpreting the study findings. First, while the FARS has reported race and Hispanic ethnicity data since 1999, two or more or multiple race categories were not added until 2019 (52, 53). As a result, the proportion of the White race category could have been overestimated before 2019. There is a significant amount of redacted, unknown race, and ethnicity information within the FARS database. Rosenberg reported that the incomplete reporting of race and ethnicity to federal agencies is a longstanding issue (54). Consequently, our results on race/ethnicity may have potentially biased our estimates. Second, the responsibility for reporting and registering death information falls to individual states (55) on which FARS relies. This can lead to state level variation in missing data or incomplete reporting of race and ethnicity information. In addition, the use of a combined “non-Hispanic/Unknown” race/ethnicity category may introduce misclassification bias, obscure heterogeneity across racial and ethnic groups. Third, we used the total population of teens aged 15–19 years as the denominator for both all-teen and teen-driver fatality rates to ensure consistency across outcomes. However, using population rather than measures such as vehicle miles traveled assumes equal driving exposure across sex and race/ethnicity groups, an assumption that is unlikely to hold. Licensure rates and driving exposure vary by sex, race/ethnicity, and over time, including during the COVID-19 period. As a result, our approach may underestimate fatality risk among licensed teen drivers and overestimate risk for groups with lower driving participation. Shifts in licensure patterns may therefore bias comparisons across groups and time periods by conflating differences in crash risk with differences in driving exposure. We acknowledge that race and ethnicity may partially reflect geographic distribution across U.S. regions, and that differences in traffic environments, policies, and infrastructure may contribute to the disparities observed. Finally, the lack of significant differences across COVID-19 periods may reflect limited statistical power, as analyses were based on only three aggregated time points using a pre-, during-, and post-pandemic indicator. Detailed jurisdiction-level data on policy restrictions or behavioral changes were not included in our model. Given these limitations, including the absence of reliable estimates of the teen driving population and potential pandemic-related impacts, our findings should be interpreted with caution (20).

## 5 Conclusion

Previous research has shown that racial and ethnic minority populations in the U.S. face a higher risk of dying in MVCs than their majority counterparts (8, 56–58). Our study supports some of these findings, indicating that male teens and American Indian or Alaska Native teens in the U.S., regardless of their vehicle occupancy status (driver, passenger, or non-driver), had the highest rate of MVC-related fatalities compared to their females and teens of other racial and ethnic groups. Additionally, Black teens are more likely to die as non-drivers in fatal crashes. Together, these results underscore the significant influence of sex, and race/ethnicity on crash-related mortality, and highlight pronounced disparities among American Indian or Alaska Native and Black male teens. Addressing these inequities requires immediate policy action and targeted investment. Our findings suggest that limited access to driver education and safe vehicles may contribute to disparities in teen MVC fatalities, highlighting the need for programs that promote equitable access to driver education, such as driving school vouchers, and provide financial support for vehicle maintenance and safety checks. Although classifications of teen drivers and race/ethnicity groups are specific to the U.S., these results have broader relevance for understanding differential risk and promoting road safety among vulnerable teen populations worldwide.

## Data Availability

Publicly available datasets were analyzed in this study. This data can be found here: in the Fatality Analysis Reporting System (FARS) FTP Site, https://www.nhtsa.gov/file-downloads?p=nhtsa/downloads/FARS/ and the American Community Survey (ACS), https://www.census.gov/programs-surveys/acs/.
